# In Vivo, In Vitro and In Silico Anticancer Activity of Ilama Leaves: An Edible and Medicinal Plant in Mexico

**DOI:** 10.3390/molecules29091956

**Published:** 2024-04-24

**Authors:** Jesica Ramírez-Santos, Fernando Calzada, Rosa María Ordoñez-Razo, Jessica Elena Mendieta-Wejebe, José Antonio Velázquez-Domínguez, Raúl Argüello-García, Claudia Velázquez, Elizabeth Barbosa

**Affiliations:** 1 Instituto Politécnico Nacional, Escuela Superior de Medicina, Sección de Estudios de Posgrado e Investigación, Plan de San Luis y Salvador Díaz Mirón S/N, Col. Casco de Santo Tomás, Mexico City 11340, Mexico; jes.ram.san@gmail.com (J.R.-S.); jesmenwej@yahoo.com (J.E.M.-W.); rebc78@yahoo.com.mx (E.B.); 2 Unidad de Investigación Médica en Farmacología, UMAE Hospital de Especialidades 2° Piso CORSE Centro Médico Nacional Siglo XXI, Instituto Mexicano del Seguro Social, Av. Cuauhtémoc 330, Col. Doctores, Mexico City 06720, Mexico; 3 Unidad de Investigación Médica en Genética Humana, UMAE Hospital Pediatría, 2° Piso, Centro Médico Nacional Siglo XXI, Instituto Mexicano del Seguro Social, Av. Cuauhtémoc 330, Col. Doctores, Mexico City 06725, Mexico; romaorr@yahoo.com.mx; 4 Instituto Politécnico Nacional, Escuela Nacional de Medicina y Homeopatía, Av. Guillermo Massieu Helguera 239, La Purísima Ticoman, Gustavo A. Madero, Mexico City 07320, Mexico; jauam14@yahoo.com.mx; 5 Departamento de Genética y Biología Molecular, Centro de Investigación y de Estudios Avanzados del Instituto Politécnico Nacional, Av. Instituto Politécnico Nacional 2508, San Pedro Zacatenco, Gustavo A. Madero, Mexico City 07360, Mexico; raularguellogarcia@yahoo.com; 6 Área Académica de Farmacia, Instituto de Ciencias de la Salud, Universidad Autónoma del Estado de Hidalgo, Km 4.5, Carretera Pachuca-Tulancingo, Unidad Universitaria, Pachuca 42076, Mexico; cvg09@yahoo.com

**Keywords:** agri-food waste, ilama leaves, *Annona macroprophyllata*, acyclic terpenoids, cancer, morphological analysis, flow cytometry, docking

## Abstract

Ilama leaves are an important source of secondary metabolites with promising anticancer properties. Cancer is a disease that affects a great number of people worldwide. This work aimed to investigate the in vivo, in vitro and in silico anticancer properties of three acyclic terpenoids (geranylgeraniol, phytol and farnesyl acetate) isolated from petroleum ether extract of ilama leaves. Their cytotoxic activity against U-937 cells was assessed using flow cytometry to determine the type of cell death and production of reactive oxygen species (ROS). Also, a morphological analysis of the lymph nodes and a molecular docking study using three proteins related with cancer as targets, namely, Bcl-2, Mcl-1 and VEGFR-2, were performed. The flow cytometry and histomorphological analysis revealed that geranylgeraniol, phytol and farnesyl acetate induced the death of U-937 cells by late apoptosis and necrosis. Geranylgeraniol and phytol induced a significant increase in ROS production. The molecular docking studies showed that geranylgeraniol had more affinity for Bcl-2 and VEGFR-2. In the case of farnesyl acetate, it showed the best affinity for Mcl-1. This study provides information that supports the anticancer potential of geranylgeraniol, phytol and farnesyl acetate as compounds for the treatment of cancer, particularly with the potential to treat non-Hodgkin’s lymphoma.

## 1. Introduction

Ilama (*Annona macroprophyllata* Donn. Sm.; Syn: *Annona diversifolia* Saff.) belongs to the Annonaceae family and is a native plant from southwestern Mexico and Central America, and it is of great importance due to the high quality of its edible fruits [1]. It is known in Mexico by several local names, including “papauce, annona blanca, ilama zapote, ilamazapotl, izlama, hilama” and “zapote de vieja”. The leaves of this plant are used in Mexican traditional medicine for the treatment of diabetes, inflammation, cancer and pain [2]. Ilama has become one of the best known, commercialized and extensively studied species in recent years considering its therapeutic potential and traditional use. The fruit of this plant is used as food, but its leaves are a waste product at the agro-industrial level [3,4].

Therefore, an efficient use of ilama leaves is required. In this context, it is important to highlight that several studies of different extracts obtained from ilama leaves have shown that they possess flavonoid glycosides, acyclic sesquiterpenoids and an aliphatic ketone. In the case of pharmacological studies, ilama leaves exhibited anxiolytic, antinociceptive, anticonvulsant, antihyperglycemic and antiepileptic effects [2,3,4,5,6].

Petroleum ether extract of leaves from *Annona macroprophyllata* and geranylgeraniol (Gg), phytol (PT) and farnesyl acetate (FA), three acyclic sesquiterpenoids obtained from this species of Annonaceae, exhibited antilymphoma activity in mice [6]. In this context, further studies are needed to examine the anticancer properties of *A*. *macroprophyllata* and its acyclic sesquiterpenoids using in vivo, in vitro and in silico approaches. This information is necessary to support their anticancer potential and elucidate their mechanism of action.

Cancer is a disease that affects a significant number of patients each year, and according to data from the World Health Organization, it is the leading cause of death worldwide; in 2020, it caused the death of 10 million people [7]. Around 19.3 million new cases of cancer have been diagnosed since then, and this is expected to increase in the coming years [6,8]. Non-Hodgkin’s lymphoma (NHL) is the most common hematologic neoplasia characterized by the abnormal development of lymphocytes, affecting the lymph nodes, spleen, bone marrow, stomach and skin. [9]. In 2020, NHL caused the death of 259,793 individuals worldwide [10]. Some of the factors of this disease are age, lifestyle, weakened immune system, acquired infections and genetic risk [11].

Some natural bioactive compounds isolated from medicinal plants have an important role as anticancer agents [12]. Approximately sixty percent of the drugs used to treat cancer were isolated from natural products. Mexico has a great wealth of plants used in traditional medicine, since most people with cancer resort to other alternatives to treat this disease, considering that chemotherapy and radiotherapy treatments are expensive. In addition, people are discouraged by the numerous side effects they produce [13].

Terpenoids are the largest class of natural products, including more than 40,000 structures, and these molecules provide benefits to humans as they are used in the fragrance, chemical and pharmaceutical industries [14]. Some terpenoids have been shown to have anticancer effects, targeting cancer initiation and progression at various stages, including those involved in cell proliferation, differentiation, cell death, angiogenesis and metastasis [14,15]. Some mechanisms of action have been demonstrated for the anticancer activity of terpenoids, such as the inhibition of NF-κB signaling, DNA damage, topoisomerase inhibition, arrest of the cycle cell, activation of apoptosis and generation of reactive oxygen species (ROS) [14,15,16]. In the case of ROS, it has been reported that elevated levels of production of ROS lead to oxidative stress, a status closely associated with apoptotic cell death. However, there is a need for studies that allow for establishing the mechanism of action of acyclic terpenes against cancer, particularly to treat non-Hodgkin’s lymphoma (NHL).

The presence and accumulation of molecular changes have been reported to be involved in the different stages of carcinogenesis in several types of cancer, including NHL [8]. Some of the biomarkers can be used as potential therapeutic targets. The Bcl-2 family has been reported to regulate apoptosis and mitochondrial events, determining whether a cell should live or die [17]. These proteins respond to signals from various forms of intracellular stress, such as DNA damage or cytokine deprivation. Two of the proteins that make up this family are Bcl-2 and Mcl-1. It is well known that Bcl-2 is overexpressed in follicular center B lymphomas due to translocation (14; 18); high levels have also been detected in chronic lymphocytic leukemia, diffuse large B-cell lymphoma and mantle lymphoma. The altered function of this proto-oncogene has been attributed to Bcl-2 promoter hypomethylation occurring in one of the Bcl-2 copies in B-cell lines, allowing malignant cells to generate resistance to apoptosis [18]. Moreover, protein myeloid cell leukemia-1 (Mcl-1) is overexpressed in multiple myeloma, acute myeloid leukemia and several hematologic cancers that make up the NHL. Mcl-1 has been shown to play an important role in cell survival and resistance to chemotherapy and radiation therapy in malignant cells [19]. In addition, Mcl-1 expression has been correlated with tumor grade and was determined to be predominant in high-grade lymphomas versus low-grade lymphomas [20]. Another possible biomarker is vascular endothelial growth factor receptor 2 (VEGFR-2); this is an enzyme belonging to the tyrosine kinase group that plays an important role in cancer growth through angiogenesis [21]. It is well known that tumor tissue needs the development of new blood vessels to supply oxygen and nutrients to new tumor cells [22]. These findings suggest continuing the challenge of searching for selective, effective and safe inhibitory molecules of some potential biomarkers for NHL.

In order to continue the search for potential anticancer agents, information on the effects of the acyclic terpenoids FA, Gg and PT obtained from ilama leaves, specifically on NHL, are reported in this work, including the induction of ROS, the induction of apoptosis, a morphological analysis of lymph nodes of mice with NHL by histology and molecular docking using proteins associated with cancer as targets, including Bcl-2, Mcl-1 and VEGFR-2.

## 2. Results

### 2.1. Antilymphoma Activity of Acyclic Terpenoids Isolated from Ilama Leaves

**Figure 1:**
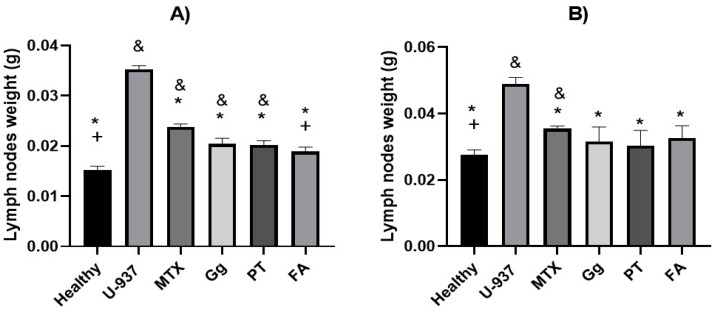
Weights (in g) of lymph nodes of female (**A**) and male (**B**) mice compared with healthy control (HC), control without treatment at 65 days (U-937), methotrexate (MTX), geranylgeraniol (Gg), phytol (PT), and farnesyl acetate (FA). Results were obtained by ANOVA one-way analysis followed by Bonferroni’s test for multiple comparisons. Data are expressed as mean ± SEM, (n = 6); * *p* < 0.05 vs. U-937, + *p* < 0.05 vs. MTX, and ^&^ *p* < 0.05 vs. healthy.

To evaluate the antilymphoma activity of the terpenoids geranylgeraniol (Gg), phytol (PT) and farnesyl acetate (FA), the weight of the axillary and inguinal lymph nodes of mice with NHL were obtained (Figure 1), and the percentages of lymph node growth inhibition were calculated at a dose of 10 mg/kg of body weight in female and male mice. Overall, the antilymphoma activities of the three terpenoids were best compared to the reference drug methotrexate (MTX). In addition, the antilymphoma activities in the female and male mice were close to AF, Gg and PT. In the female mice, the lymph node growth inhibition was 81.6 ± 3.89%, 74.8 ± 6.38%, 73.9 ± 5.52% and 57.0 ± 6.62% for FA, PT, Gg and MTX, respectively. In the case of the male mice, the lymph node growth inhibition was 76.20 ± 6.98%, 88.6 ± 4.83%, 81.3 ± 8.43% and 63.3 ± 3.84% for FA, PT, Gg and MTX, respectively.

### 2.2. Effect of Geranylgeraniol, Phytol and Farnesyl Acetate Isolated from Ilama Leaves on Apoptosis and Necrosis in U-937 Cells

**Figure 2:**
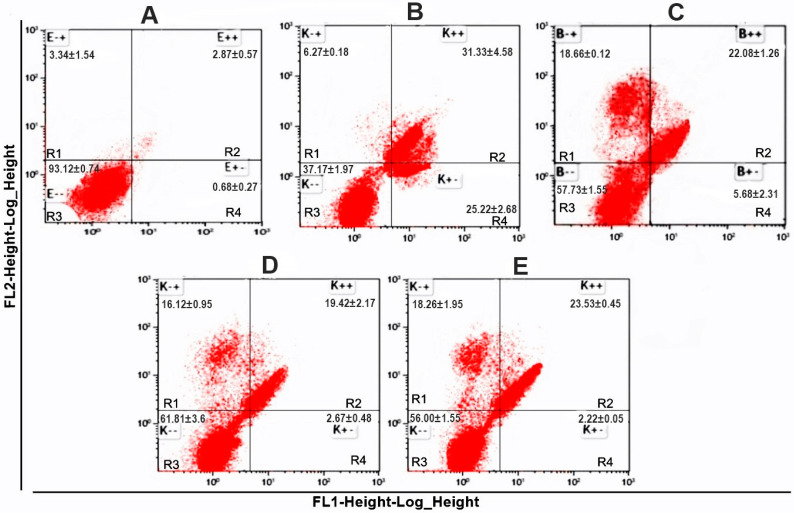
Apoptotic and necrotic effects of Gg, PT and FA. U-937 cells were exposed to vehicle 0.02% dimethyl sulfoxide (DMSO, untreated control) (**A**), methotrexate (CC_50_ 0.243 µM, MTX) (**B**), geranylgeraniol (CC_50_ 0.395 µM, Gg) (**C**), phytol (CC_50_ 0.296 µM, PT) (**D**) and farnesyl acetate (CC_50_ 0.275 µM, FA) (**E**) and were incubated with annexin-V-FITC/PI and analyzed by flow cytometry. R1 = necrosis, annexin-V-FITC-negative/PI-positive (E−+, K−+ and B−+); R2 = late apoptosis, annexin-V-FITC-positive/PI-positive (E++, K++ and B++); R3 = viable cells, annexin-V-FITC-negative/PI-negative (E−−, K−− and B−−); R4 = early apoptosis, annexin-V-FITC-positive/PI-negative (E+−, K+− and B+−).

Once the antilymphoma activity of the terpenoids were determined, the induction of cell death by apoptosis or necrosis was evaluated (Figure 2). The apoptosis detection analysis by flow cytometry showed, in early apoptosis (quadrant R4), that all the terpenoids showed weak effects on the U-937 cells; in contrast, MTX generated an increase in the percentage of cell death in early apoptosis with a value of 25.22 ± 2.68% compared to the vehicle DMSO (untreated control). In the case of late apoptosis (quadrant R2), the percentages of cell death in late apoptosis were 22.08 ± 1.26%, 19.42 ± 2.17% and 23.53 ± 0.45% for Gg, PT and FA, respectively. Their effects were like that of MTX (31.33 ± 4.58%) compared to the vehicle (DMSO). Finally, for necrotic cells or cells that did not die by apoptosis (quadrant R1), the results showed that Gg, PT and FA induced cell death of 18.66 ± 0.12%, 16.12 ± 0.95% and 18.26 ± 1.95%, respectively. In contrast, MTX showed a weak effect. The results obtained showed that the terpenoids geranylgeraniol, phytol and farnesyl acetate induced programmed death in the U-937 cells by late apoptosis and necrosis. In the case of MTX, it induced death in the U-937 cells by early and late apoptosis.

### 2.3. Effect of Geranylgeraniol, Phytol and Farnesyl Acetate Isolated from Ilama Leaves on Induction of Generation of ROS in U-937 Cells

**Figure 3:**
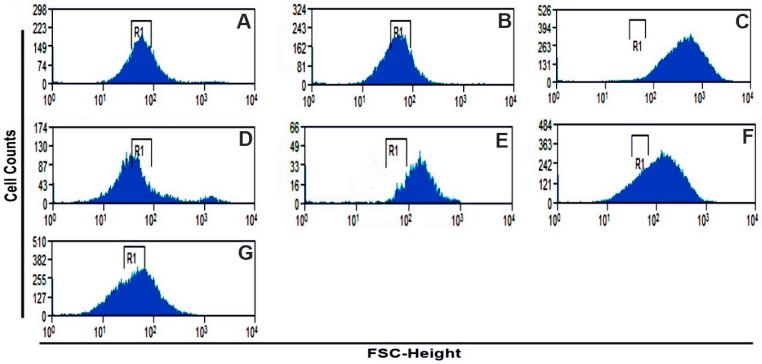
Geranylgeraniol and phytol produced an increase in the generation of ROS. U-937 cells were cultured in DMEM medium (**A**) or exposed to vehicle 0.02% dimethyl sulfoxide (DMSO) (**B**), 150 µM hydrogen peroxide (H_2_O_2_) (**C**), methotrexate (CC_50_ 0.243 µM) (**D**), geranylgeraniol (CC_50_ 0.395 µM) (**E**), phytol (CC_50_ 0.296 µM) (**F**) or farnesyl acetate (CC_50_ 0.275 µM) (**G**) and were incubated with 2′-7 dichloro-dihydrofluorescein diacetate, and the fluorescence of reactive oxygen species (ROS) was analyzed by flow cytometry. *x*-axis: fluorescence scatter (FSC); *y*-axis: cell counts.

Once the induction of late apoptosis was demonstrated by the acyclic terpenoids Gg, PT, and FA, we evaluated their capability of inducing the production of ROS in the U-937 cells, considering that the production of ROS in physiologic and pathological cases may be associated with the induction of apoptosis [6]. The results showed that the terpenoids Gg and PT induced a significant increase in ROS production, with values of 58.7 and 31.5% (Table 1) of the mean fluorescence shift (Figure 3E,F). Their effects were less active in comparison with that of hydrogen peroxide (H_2_O_2_) (Figure 3C), which was used as a positive control and induced a significant increase in the mean fluorescence displacement, with a value of 71.30%. In contrast, FA and MTX did not produce any change in the florescence displacement and therefore, at the doses tested, did not have the ability to generate the production of ROS (Figure 3D,G). These results suggest that the antitumoral properties, as inductors of apoptosis, of Gg and PT may be mediated by the generation of ROS.

### 2.4. Morphological Analysis Using Histology of Axillary Lymph Nodes

**Figure 4:**
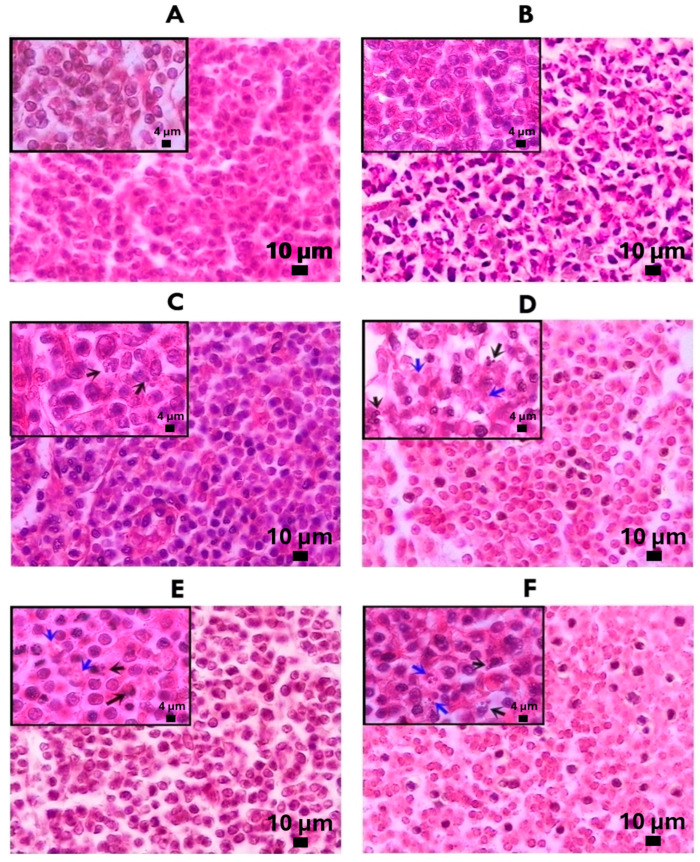
Representative photomicrographs with H&E staining of transversal axillary lymph node. Paracortex area (7 μm) (40X). Healthy control (**A**); U-937 (**B**), methotrexate (**C**), phytol (**D**), geranylgeraniol (**E**) and farnesyl acetate (**F**). Photomicrographs (7 μm) (100X) show cells in apoptosis (black arrows) and necrotic cells (blue arrows).

The histological analysis of photomicrographs of the axillary lymph nodes of male mice stained with hematoxylin and eosin (H&E) is shown in Figure 4. The axillary lymph nodes in the healthy control group showed the presence of follicles with their germinal center, so their structure was normal. Meanwhile, in the U-937, Gg, PT, FA and MTX groups, they did not show follicles; therefore, the morphological analysis indicated a diffuse-type architecture as part of the tumor process. In the paracortex area, U-937 consisted of medium-sized and large spindle-shaped and round cells. The nucleus was irregular, eosinophilic and moderately eccentric, and there was a moderately stained cytoplasm. The degree of cellular pleomorphism was generally pronounced. In addition, an increase in connective tissue was observed due to the presence of eosin staining on fibroblasts, collagen fibers and fibrils. While MTX showed a reduction in pleomorphic and spindle cells compared to U-937, it also showed a reduction in strongly stained hematoxylin cells. On the other hand, Gg, PT and FA also showed a reduction in pleomorphic, spindle and strongly hematoxylin-stained cells compared to U-937 and showed a reduction in the presence of connective tissue. At a higher magnification, it was observed that Gg, PT, FA and MTX produced pyknosis, cell contraction, compaction of the nucleus, chromatin condensation and blisters on the cell surface in some cells; these characteristics correspond to apoptotic cells. In addition, Gg, PT and FA generated focal necrosis, and some cells also showed a loss of cytoplasmic membrane integrity and an exit of the cell contents into the interstitium.

### 2.5. Molecular Docking Studies of Geranylgeraniol, Farnesyl Acetate and Phytol

**Figure 5:**
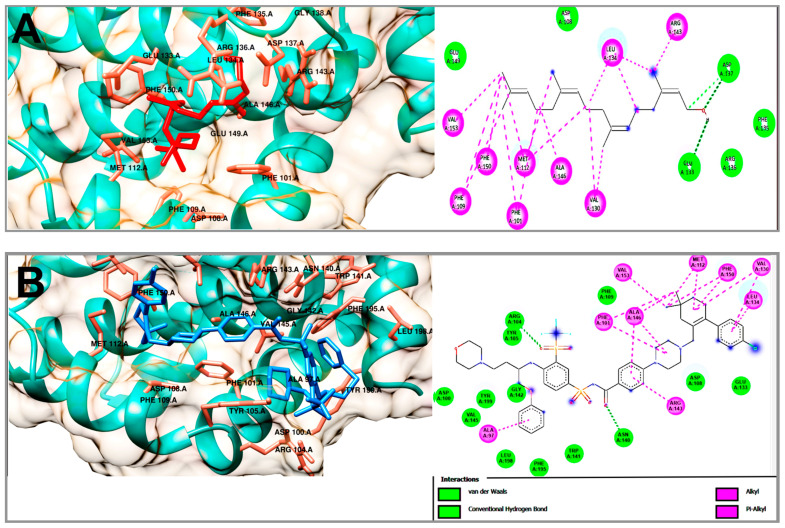
Molecular docking of the acyclic terpenoid geranylgeraniol (**A**) and navitoclax (**B**) bound to Bcl-2.

**Figure 6:**
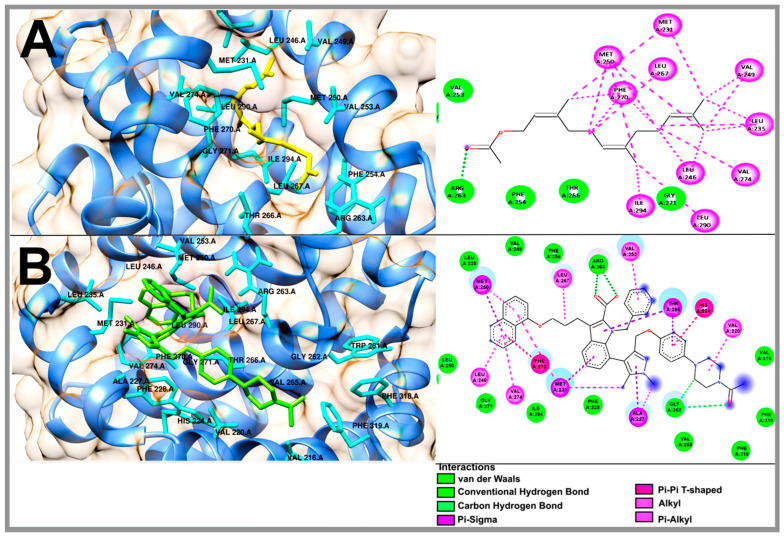
Molecular docking of the acyclic terpenoid farnesyl acetate (**A**) and 9EA (**B**) bound to Mcl-1.

**Figure 7:**
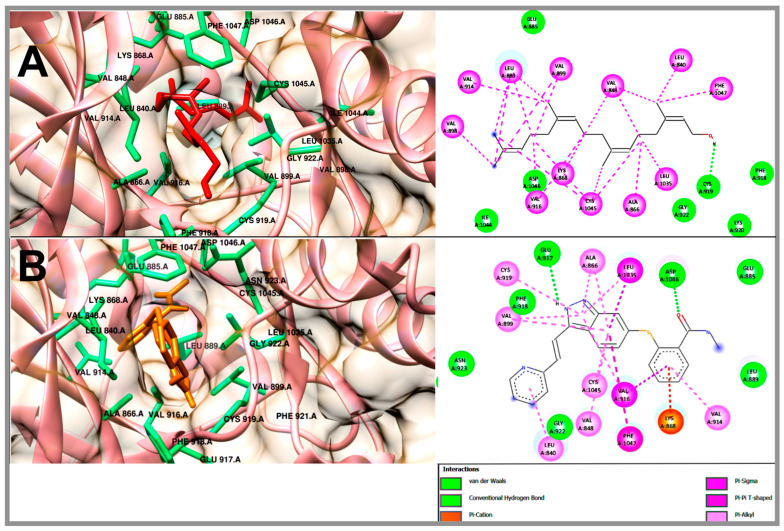
Molecular docking of the acyclic geranylgeraniol terpenoid (**A**) and axitinib (**B**) bound to VEGFR-2.

In order to understand the antilymphoma properties of the acyclic terpenoids Gg, PT and FA and how they affect U-937 cells, we decided to study three potential targets associated with cancer, including Bcl-2, Mcl-1 and VEGFR-2. In this context, Bcl-2 and Mcl1 are two proteins that are overexpressed in NHL [18,19,20,23]. In addition, the selected targets were also consulted in the DisGeNET (https://www.disgenet.org/ (accessed on 20 february 2024)) and GeneCards (https://www.genecards.org/) databases (accessed on 15 March 2024). The results obtained from the molecular docking study are shown in Table 2. The binding energies to FA, PT and Gg obtained for Bcl-2 were −6.47, −6.91 and −7.33 kcal/mol, respectively (Table 2). The Bcl-2 inhibitor used was navitoclax (ABT-263), which showed a binding energy of −12.54 kcal/mol. In addition, Gg, PT and FA shared some nonpolar interactions with navitoclax in the hydrophobic binding groove of Bcl-2, including Phe 101, Phe 109, Met 112 and Ala 146 (Figure 5 and Figure S1 in the Supplementary Materials) [24,25]. In the case of Mcl-1, the acyclic terpenoids Gg, PT and FA showed binding energies of −6.46, −6.35 and −6.69 kcal/mol, respectively. The binding affinity to the inhibitor 9EA (7-(3-{[4-(4-acetylpiperazin-1-yl)-phenoxy] methyl}-1,5-dimethyl-1H-pyrazol-4-yl)-3-{3-[(naphthalen-1-yl) oxy] propyl}-1-[(pyridin-3-yl) methyl]-1H-indole-2-carboxylic acid) was −10.77 kcal/mol (Table 2). In addition, the three terpenoids shared nonpolar interactions with 9EA, including Met231, Leu235, Leu246, Val249, Met250, Leu267, Phe270, Leu290 and Ile294 (Figure 6 and Figure S2 in the Supplementary Materials). These residues are part of the binding pocket for Mcl-1 [26]. In this specific case, PT and FA shared an interaction on Arg 263 with 9EA, an essential residue for Mcl-1 activity. Finally, for VEGFR-2, the results of the binding energies were −6.3, −5.98 and −5.82 kcal/mol to Gg, PT and FA, respectively. The inhibitor used for this study was axitinib, and it showed binding energy of −8.57 kcal/mol (Table 2). The acyclic terpenoids shared three H-binding residues with axitinib, including Phe918, Gly 922 and Asp 1046 (Figure 7 and Figure S3 in the Supplementary Materials); among these residues, Asp 1046 is part of the DFG sequence that plays an important role in the ATP binding and activation loop [22]. Also, the acyclic terpenoids shared all nonpolar interactions with axitinib [27].

## 3. Discussion

In the year 2020, non-Hodgkin’s lymphomas ranked eleventh in terms of mortality worldwide and fourth in Mexico [7]. For this reason, it is important to search for new anticancer agents with greater efficacy and fewer side effects. In this sense, obtaining natural products isolated from medicinal plants with antitumor potential could be important in obtaining agents for the treatment of various human diseases, including cancer.

Ilama is an important edible fruit cultivated in the southwestern Mexico and Central America, but its leaves are considered wasteful. Several researchers have explored ilama leaves as a source of antihyperglycemiants and anticancer agents. It has been reported that ilama leaves contain flavonoid glycosides, sesquiterpenoids and an aliphatic compound [2,6].

In this work, the anticancer effects of geranylgeraniol (Gg), phytol (PT) and farnesyl acetate (FA) isolated from *Annona macroprophyllata* [6] were evaluated using in vivo, in vitro and in silico approaches. The cytotoxic activity on U-937 cells was tested using flow cytometry to determine the type of cell death and production of reactive oxygen species (ROS). In addition, a morphological analysis of the lymph nodes obtained from Balb-c mice with NHL and a molecular docking study using proteins related with cancer, namely, Bcl-2, Mcl-1 and VEGFR-2, were performed.

First, the antilymphoma activities of Gg, PT and FA were determined at a dose of 10 mg/kg. The results showed that the three acyclic terpenoids obtained had close antilymphoma activities, and the lymph node weight in the female and male mice was significantly decreased compared to the untreated groups (Figure 1). FA showed the best inhibition of axillary and inguinal lymph node growth in the female mice, and PT had the best antilymphoma activity in the male mice, with values of 81.6 ± 3.89% and 88.6 ± 4.83%, respectively. These results are consistent with the results obtained from our previous work [6] and with other reports on the antitumor activities of some terpenoids on colon, hematologic, brain, prostate, bladder, gastric, lung and bone cancers [28,29,30]. The growth inhibition effect of MTX on the lymph nodes was lower in both sexes of mice compared to that of Gg, PT and FA, with values of 57 ± 6.62% of inhibition in females and 63.3 ± 3.84% of inhibition in males at a dose of 1.25 mg/kg. In this context, it is important to highlight that at doses > of 1.25 mg/kg, MTX was toxic and caused death in the animals. This correlates with the toxicological reports of MTX, since the adverse effects it produces are well known, such as myelosuppression, with involvement of all cell series, renal failure due to the involvement of renal tubules and the fact that it can become neurotoxic; therefore, patients who receive this drug should have good dosage management [31,32].

Once the antilymphoma activities of the acyclic terpenoids Gg, PT and FA were determined using a model of mice inoculated with U-937 cells, we focused on determining the type of cell death that the terpenoids induced. The detection of apoptosis was analyzed by flow cytometry using annexin V-FITC staining and propidium iodide (PI) as a fluorescent dye; the first is a marker that binds to phosphatidylserine in the outer cell membrane, and the second is a fluorescent dye that stains mainly nucleic acids, so it is an indicator of direct damage to the cell membrane. The apoptosis analysis (Figure 2) showed, in early apoptosis (quadrant R4), that Gg, PT and FA exhibited weak effects on the U-937 cells; in contrast, MTX generated an increase in the percentage of cell death, with a value of 25.22 ± 2.68% compared to all the acyclic terpenoids. In late apoptosis (quadrant R2), the percentage of cell death of all the acyclic terpenoids were like that of MTX. In the case of necrosis (quadrant R1), the results showed that Gg, PT and FA induced cell death from 18.66 ± 0.12%, 16.12 ± 0.95% and 18.26 ± 1.95%, respectively. The results obtained showed that at 24 h of exposure with the terpenoids geranylgeraniol, phytol and farnesyl acetate induced programmed death in the U-937 cells by the induction of late apoptosis and necrosis. Therefore, the antilymphoma activity of the acyclic terpenoids Gg, PT and FA may be mediated in part by the induction of late apoptosis and necrosis. Our results are in agreement with the results reported by other authors, where PT induced the activation of caspase 9 (intrinsic apoptosis) and produced the activation of TNF-related receptors of the inducer ligand TRAIL, DR4 and DR5, leading to the activation of caspase 8 (extrinsic apoptosis) [31]. In addition, in A549 cells (lung carcinoma), hepatocellular carcinomas, including Huh7 (hepatic carcinoma) and HepG2 (hepatocellular carcinoma) cells, were associated with caspase-dependent apoptosis (9 and 3) and epithelial mesenchymal transition signaling [32]. Gg suppressed the viability of DU145 (human prostate carcinoma) and DLD1 (colon cancer) cells, generating cell cycle arrest in the G phase. Also, it affected HL-60 (human promyelocytic leukemia cells), K562 (chronic myelogenous leukemia), Molt-3 (acute lymphoblastic leukemia) and COLO320 (adenocarcinoma of the colon) by DNA damage, and these processes eventually led to apoptosis through the activation of caspases (3, 8 and 9) and caused the release of cytochrome C [33,34,35,36]. In the case of MTX, the results obtained from the induction of apoptosis on the U-937 cells are in agreement with some studies on its inhibition effects on the enzyme dihydrofolate reductase, thus preventing the conversion of dihydrofolate to tetrahydrofolate. Tetrahydrofolate is essential for the synthesis of purines and thymidine. This blockade could lead to the generation of apoptosis by inhibiting DNA synthesis and consequently stopping cell division and protein production [32,37].

Once the induction of late apoptosis and necrosis were demonstrated by the terpenoids Gg, PT and FA, we decided to evaluate their capability of inducing the production of ROS in U-937 cells, considering that the production of ROS may be associated with apoptosis [6]. The results showed that Gg and PT induced an increase in ROS (Figure 3E,F) in the U-937 cells, and in the case of FA and MTX, they were inactive. These results suggest that the antilymphoma effects of Gg and PT may be associated with their capacity to induce late apoptosis, necrosis and ROS [12,13,14,15]. PT caused increased ROS levels in human gastric adenocarcinoma cells, A549 cells (lung carcinoma) and A431 cells (squamous cell carcinoma) [37,38,39]. In addition, Gg produced increased levels of superoxide in TYK-nu (undifferentiated carcinoma) cells [40]; all previous reports have confirmed that the production of ROS leads to processes that trigger cell apoptosis. It is well known that an increase in ROS could lead to a depletion of antioxidant proteins and thus lead to cell death. It has been reported that ROS can lead to apoptosis initiated by intrinsic or extrinsic signaling mediated by the death receptor pathway [41,42]. The binding of ligands to death receptors triggers the caspase activation of caspase initiator 8, resulting in the cleavage of caspase 3 and the Bcl-2 protein to tBid, which translocates to the mitochondria, resulting in the release and translocation of cytochrome C. In addition, some evidence indicates that an increase in ROS generates damage to the mitochondrial electron transport chain, resulting in the release of cytochrome C, which triggers the mitochondrial apoptotic process [42].

In the case of the histological studies of the axillary lymph nodes, it was revealed that the healthy mice used as the control group had one or more lymphoid nodes, whereas in contrast, the mice of the U-937, Gg, PT and FA groups did not present any nodules, so they showed a diffuse architecture. In the paracortex area, the U-937 group showed a diffuse pattern with medium and large pleomorphic and spindle cells; these features represent tumors with a histiocytic architecture [43]. Also, an increase in connective tissue was observed, as demonstrated by the highly marked staining on the reticular mesh made up of stellate fibroblast reticular cells and their reticular fibers [44]. However, after the administration of the Gg, PT and FA treatments, the lymph nodes showed a reduction in spindle and pleomorphic cells in the paracortex compared to the U-937 group in addition to a decrease in connective tissue.

Using the 100X objective, it was observed that the MTX, Gg, PT and FA treatments produced pycnosis, nuclear compaction, chromatin condensation and the formation of blisters on the cytoplasmic surface on some cells in the paracortex area; these characteristics are associated with the morphology of apoptotic cells [45]. In addition, the Gg, PT and FA groups showed the presence of cells with the morphological characteristics of necrotic cells, since some cells lost the integrity of the cytoplasmic membrane and their cell content was released into the interstitium. Also, the presence of inflammatory cell infiltrates, such as plasma cells, eosinophils and histiocytes, was observed; this histological morphology has been directly related to necrotic processes [46]. These morphological observations using H&E staining agree with the results obtained for the induction of late apoptosis and necrosis by Gg, PT and FA in the flow cytometry experiments.

In order to understand and how the terpenoids Gg, PT, and FA can affect the three potential molecular targets Bcl-2, Mcl-1 and VEGFR-2, considering that these proteins are associated with cancer, a docking study was carried out, and the effects were compared against those of axitinib, 9EA and navitoclax, which were used as reference inhibitors.

The Bcl-2 and Mcl-1 proteins belong to the Bcl-2 family of proteins, which are key to cell survival. Some findings have determined that these proteins have an important role in the regulation of intrinsic apoptosis by releasing cytochrome C from the mitochondria through the alteration of mitochondrial permeability [47]. For example, the Bax and Bak proteins function by forming homo- or heterodimeric pores in the outer mitochondrial membrane that trigger proteolytic enzymes (caspases) that cause cell demolition [48]. However, it has been reported that the overexpression of Bcl-2 and Mcl-1 in various hematologic cancers, including NHL, may lead to dysregulation of the apoptosis rate, benefiting cancer progression [18,19,20]. In recent years, the search for molecules that bind to the BH3 domain on Bcl-2 and Mcl-1 has been proposed, as it could be promising for the therapy of cancers and some types of NHL with the overexpression of these proteins [49].

The theoretical results obtained from the molecular docking analyses of Gg, PT and FA on Bcl-2 showed close binding energies of −7.33, −6.47 and −6.91 kcal/mol, respectively. These energies were weaker compared to the binding energy obtained from navitoclax. The navitoclax inhibitor has been reported to be highly effective in patients with chronic lymphocytic leukemia; thus, it was used as a control to compare its binding affinity against that of the acyclic terpenoids [25]. In addition, some of the residues reported with importance for antiapoptotic activity that are part of the hydrophobic groove of Bcl-2 [24,25] were interactions that were shared between navitoclax and the three terpenoids, and these amino acids were Phe 101, Phe 109, Met 112 and Ala 146. Therefore, we infer that Gg, PT and FA could have a sensitizer role for Bcl-2, that is, it could neutralize the antiapoptotic effect by binding at the binding site of the hydrophobic groove, also called the hydrophobic pockets P2 and P4.

In the case of Mcl-1, it has a binding pocket lined with numerous nonpolar side chains and that is much deeper and larger compared to Bcl-2 [50], so we inferred that Gg, PT and FA could obtain more favorable energies with respect to Bcl-2. However, the binding energies were close to the data obtained for Bcl-2 but were closer to its co-crystallized ligand. Overall, Gg, PT and FA obtained close binding energies of −6.46, −6.35 and −6.69 kcal/mol, respectively, but they were lower compared to their co-crystallized ligand 9EA. Most of the interactions of Gg, PT and FA with Mcl-1 were of a nonpolar type. Meanwhile, one of the amino acids that was shared by 9EA, PT and FA is of importance for Mcl-1 activity [30].

Another biomarker of importance in NHL is VEGFR-2, a protein that makes up receptor tyrosine kinase (RTK), has a role in the formation of new blood vessels (angiogenesis) and is reported to be a potential target for the suppression of cancer growth and metastasis. The results on VEGFR-2 showed that Gg, PT and FA showed close binding energies of −6.3, −5.98 and −5.82 kcal/mol, respectively. All three terpenoids and axitinib shared interactions with the key amino acid Cys 919 in the front pocket, located in the hinge area of the VEGFR-2 binding site, and with Asp 1046, a residue that integrates the DFG motif and is key to ATP binding [32]. It was inferred that Gg showed the best bonding pose with respect to PT and FA; it preserved and shared with axitinib the interaction with the key residue Glu 885, which is located in the αC helix and is also part of the DFG motif in the binding loop.

It remains to be seen whether the theoretical interactions with the evaluated molecules produce the same inhibition effects and give greater certainty about the potential therapeutic effects of Gg, PT and FA. The findings of our work support that these molecules could be a weapon in the arsenal of compounds with multiple therapeutic targets to suppress NHL. In this context, additional experiments are in progress, including a Western blot analysis to validate the results obtained in the docking study.

## 4. Materials and Methods

### 4.1. Preparation of Petroleum Ether Extract of Leaves from A. macroprophyllata

**Figure 8:**
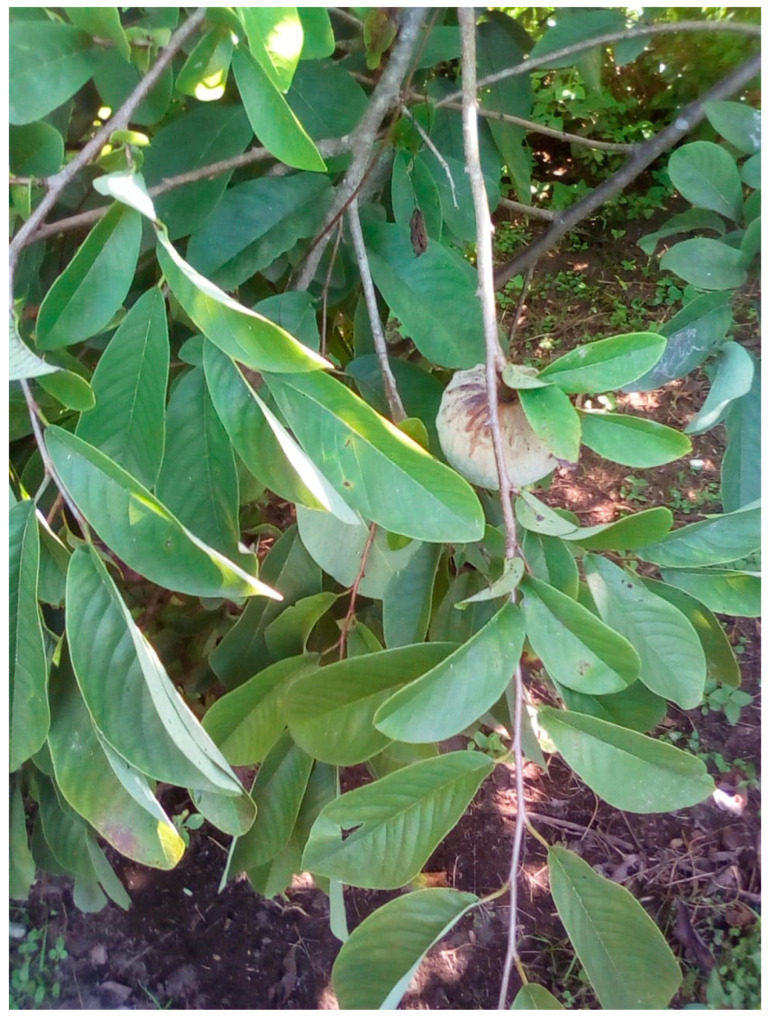
Annona macroprophyllata Donn. Sm.

*A. macroprophyllata* leaves (Figure 8) were collected from Metapa de Domínguez, Chiapas, Mexico (14°50′00″ N 92°11′00″ W). The plant material was identified by M. C. Santiago Xolalpa of the IMSSM Herbarium of the Mexican Institute of Social Security (IMSS) (Alcaldia Cuahutemoc, Cd. Mx.), with the number of specimen corresponding to 16,248. The extract was prepared by macerating the leaves at room temperature and using the solvent petroleum ether. The macerated extract was filtered and vacuum-concentrated using a rotary evaporator to yield 37.89 g of dry extract.

### 4.2. Isolation of Geranylgeraniol, Phytol and Farnesyl Acetate

**Figure 9:**
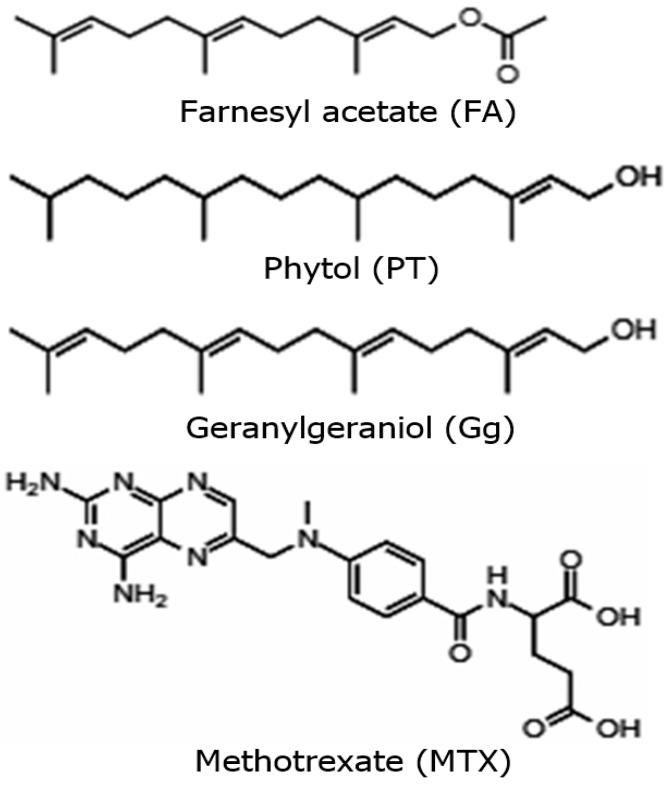
Structures of farnesyl acetate, phytol, geranylgeraniol and methotrexate.

Geranylgeraniol, phytol and farnesyl acetate (Figure 9) were isolated from the petroleum ether extract of the leaves from *A. macroprophyllata*. The procedure of its isolation and identification is described in our previous study [6]. The terpenoids were isolated and purified by preparative TLC (silica gel 60F-254 Merck, hexane-EtOAc, 85:15). For its identification, GC–MS analysis was conducted using an Agilent GC-MDS device (Agilent 220 Technologies, Wilmington, DE, USA) and the MassHunter Workstation software, version B.07.05. The terpenoids were identified using their mass spectra compared to the NIST mass spectral libraries. A comparison of retention factors with authentic sigma samples was also performed by thin-layer chromatography. To confirm the identification, the compounds were characterized by spectroscopic methods (NMR ^1^H and ^13^C).

### 4.3. Animals

Male and female mice of the Balb/c strain were kept under controlled conditions and with 12 h periods of light–dark at 22 ± 2 °C; feeding and water availability was provided ad libitum. The animals were provided by the IMSS. The approval of the experimentation procedures was evaluated by the Bioethics Committee of the Specialty Hospital of the National Medical Center “Siglo XXI”, the registration number corresponded to R-2020-3601-186 and, they were carried out under the guidelines of the Mexican official standard NOM 0062-ZOO-1999, entitled Technical Specifications for the Production, Care and Use of Laboratory Animals [51].

#### Antilymphoma Activity

To evaluate the antilymphoma activity, 1 × 10^6^ U-937 cells were injected intraperitoneally into the male and female Balb/c mice (20 ± 5 g). This procedure was performed according to the method of Calzada et al. [52]. Six groups of female mice and six groups of males (*n* = 6) were randomly formed. The healthy group (healthy, tween 80, 2% *v*/*v* in water), negative control (U-937) and positive control was treated with the reference drug methotrexate (MTX 1.25 mg-kg-1), and the groups in which the terpenoids geranylgeraniol (Gg), phytol (PT) and farnesyl acetate (FA) were evaluated were treated at a dose of 10 mg/kg. After cell inoculation, the animals were kept under observation for the next 28 days. At day 29, the treatments were administered for 9 days; the administration of each treatment was performed orally using an esophageal cannula. Weight and survival were recorded. Over the next 28 days, we recorded the weight, behavior and survival of the animals. Finally, on day 65, the left- and right-axillary and inguinal lymph nodes were removed and weighed. The antilymphoma activity was determined by comparing the total lymph node weight of each group against the negative control (U-937), and the percentage of inhibition was calculated.

### 4.4. Cell-Based Assay

#### 4.4.1. Culture

The cell line U-937 was provided by UIM en Genética Humana del Hospital de Pediatría de CMN S XXI, IMSS (ATCC: CRL 1593.2, Middlesex, UK). For its propagation two million cells were used and seeded in RPMI 1640 medium (Roswell Park Memorial Institute, Buffalo, NY, USA) supplemented with 2 mM L-glutamine, 10% *v*/*v* fetal bovine serum (Thermo, Waltham, MA, USA), 100 mM 1% sodium pyruvate, and 1% penicillin/streptomycin. The cells were kept in an atmosphere with a concentration of carbon dioxide (CO_2_) at 5% at 37 °C.

#### 4.4.2. Annexin V-FITC/IP Staining

We counted 5 × 10^5^ U-937 cells that were exposed for 24 h to the following CC_50_ values of the studied compounds obtained in our previous work [6]: Gg (CC_50_ 0.39 μM), PT (CC_50_ 0.27 μM), FA (0.29 μM) and MTX (CC_50_ 0.24 μM). Programmed cell death was determined by flow cytometry using a commercial kit (Bio Vision, Waltham, MA, USA), a conjugate of anti-annexin V-isothiocyanate fluorescein antibodies (FITC) and propidium iodide as cell markers. After 24 h of incubation with the terpenoids, the cells were washed with 500 μL of PBS and resuspended in 100 μL of PBS containing 5 μL of annexin V and PI and then incubated for 5 min at room temperature in the dark. The fluorescence was analyzed and quantified in 20,000 cells in three independent experiments using a FACSCalibur flow cytometer (Becton Dickinson^TM^, Franklin Lakes, NJ, USA).

#### 4.4.3. Measurement of Intracellular ROS

The possible production of ROS in the U-937 cell line was determined using a commercial kit according to the manufacturer’s instructions (Image-ITTM Live Green ROS Detection kit; Life Technologies, Carlsbad, CA, USA). The ROS were detected based on the 2′7′dichlorofluorescin di-acetate (DCF) oxidation to a 2′7′dichlorofluorescin assay. A total of 5 × 10^5^ cells were exposed for 24 h to Gg, PT, FA and MTX at the CC_50_ value. DMSO was used as a solvent control to rule out that the increase in ROS levels was due to its effect on cells. The positive control of ROS production used was hydrogen peroxide (100 μM), and the negative control was without any treatment. Fluorescence intensity was counted in 20,000 cells in three independent experiments using a FACSCalibur flow cytometer (Becton Dickinson^TM^).

### 4.5. Histology of Axillary Lymph Nodes

For the histological analysis of the axillary nodes, saline washes were performed after removal, and the samples were fixed with 4% PFA and then washed with double-distilled water. Subsequently, they were dehydrated using a battery of alcohols of increasing concentrations, and paraffin was included. The paraffin blocks were sectioned using a microtome Leica RM 2125RTS (Leica Biosystems, Deer Park, IL, USA), and 7 μm thick sections were obtained. Staining was performed with H&E staining [53] (Nikon Instruments Inc., Melville, NY, USA) with a SONY Exwave HAD digital color video camera (Sony, Electronics Inc., Paramus, NJ, USA).

### 4.6. Molecular Docking Studies

To perform the theoretical experiments of molecular docking, the targets were selected considering the possible interaction of the terpenoids studied (Gg, PT and FA) and their possible pharmacological targets obtained from the Swiss Target Prediction (http://www.swisstargetprediction.ch (accessed on 20 March 2024) database. This allowed us to acquire the potential targets. Targets whose credibility value was 0 were discarded. Subsequently, potential targets for NHL were acquired using the two databases DisGeNET (https://www.disgenet.org/ (accessed on 20 March 2024) and GeneCards (https://www.genecards.org/ (accessed on 20 March 2024). Based on the results obtained from the two databases, three common objectives were selected for the three terpenoids that were of importance for the therapeutics of NHL.

Then, the chemical structures of the ligand farnesyl acetate (CID: 638500), phytol (CID: 5280435), geranylgeraniol (CID: 5281365), 1XJ or navitoxlax (CID: 24978538), 9EA (CID: 68938909) and AXI or axitinib (CID: 6450551) were retrieved from the chemical library PubChem (https://pubchem.ncbi.nlm.nih.gov/) (accessed on 10 August 2023), and these were subsequently optimized and subjected to energy and geometric minimization using the Avogadro software [54]. Bcl-2 (RCSB, PDB ID: 4LVT), Mcl-1 (RCSB, PDB ID: 5VKC) and VEGFR-2 (RCSB, PDB ID: 4AG8) were used as the study objectives. These was retrieved from the Protein Data Bank (http://www.rcsb.org/) (accessed on 10 August 2023), removing total water molecules and ions that were not necessary for their catalytic activity to preserve the entire protein. All polar hydrogen atoms were added, ionized in a basic environment (pH = 7.4) and assigned Gasteiger charges. The calculated output topologies from the previous steps were used as the input files for the coupling simulations.

The molecular docking experiments were carried out using the Autodock 4.2 software [55], and the search parameters were as follows: a grid base procedure was used to generate the affinity maps, delimiting a grid box of 90 × 90 × 90 Å^3^ in each coordinate space with a grid point spacing of 0.375 Å; the Lamarckian genetic algorithm was used as a scoring function with a random initial population of 100 individuals and a maximum number of energy evaluations of 1 × 10^7^ cycles; and the analysis of the interactions in the enzyme/inhibitor complex was visualized with the PyMOL software (The PyMOL Molecular Graphics System, Ver 2.0, Schrödinger, LLC, DeLano Scientific, San Carlos, CA, USA). The validation of the molecular docking was carried out by re-docking each co-crystallized ligand (navitoclax, 9EA and axitinib) in the receptors. The lower-energy posture of the co-crystallized ligand was superimposed, and it was observed if it maintained the same binding position. The RMSD was calculated, and a reliable range within 2 Å was reported.

### 4.7. Statistical Analysis

The results were expressed as mean ± standard error of six measurements. The results were analyzed using the GraphPad Prisma version 5 program (GraphPad software, San Diego, CA, USA), performing one-way ANOVA and performing multiple comparison tests using Bonferroni’s test, with a value of *p* < 0.05 indicating that there were significant differences between the study groups.

## 5. Conclusions

The in vivo, in vitro and in silico results suggest that the anticancer properties, specifically the antilymphoma activities, of geranylgeraniol, phytol and farnesyl acetate are mediated in part by the induction of late apoptosis and necrosis. Geranylgeraniol and phytol produced an increase in ROS, which confirms that they may lead to the apoptosis of cancer cells. In addition, the theoretical experiments suggest that the three terpenoids could act on the Bcl-2 and Mcl-1 proteins. These molecules may also be involved in inhibiting the development of angiogenesis by binding to the active site of VEGFR-2. These results support that these terpenoids could be potential agents for the treatment of non-Hodgkin’s lymphoma associated with U-937 cells. Also, agri-food wastes such as ilama leaves may be a source of potential anticancer agents, specifically for the treatment of NHL.

## Figures and Tables

**Table 1:** Reactive oxygen species generation results. The percentages of cells with displace fluorescence are indicated.

Sample	Mean Fluorescence Displacement (%)
U-937 cells	-
DMSO	5.11
H_2_O_2_	71.30 *
Geranylgeraniol (Gg)	58.7 *
Phytol (PT)	31.5 *
Farnesyl acetate (FA)	5.36
Methotrexate (MTX)	6.06

Data are expressed as mean ± SEM of three independent experiments. Geranylgeraniol (CC_50_ 0.395 µM) and phytol (CC_50_ 0.296 µM) showed a higher production of reactive oxygen species compared to U-937. * *p* < 0.05 vs. DMSO.

**Table 2:** Interactions of farnesyl acetate (FA), phytol (PT) and geranylgeraniol (Gg) with amino acid residues on the binding sites of Bcl-2, Mcl-1 and VEGFR-2.

Compound	Bcl-2			
	ΔG (kcal/mol)	H-BR	NPI	RMSD
Geranylgeraniol	−7.33	Asp 108, Glu 133, Phe 135, Arg 136, Asp 137, Glu 149	Phe 101, Phe 109, Met 112, Val 130, Leu 134, Arg 143, Ala 146, Phe 150, Val 153	-
Phytol	−6.91	Asp 108, Glu 133, Phe 135, Asp 137, Phe 150	Phe 101, Phe 109, Met 112, Val 130, Leu 134, Arg 143, Ala 146, Val 153	-
Farnesyl acetate	−6.47	Asp 108, Phe 135, Arg 136, Asp 137, Arg 143, Phe 147, Glu 149	Phe 101, Phe 109, Met 112, Val 130, Leu 134, Ala 146, Val 153	-
Navitoclax (ABT-263)	−12.54	Asp 100, Arg 104, Tyr 105, Asp 108, Phe 109, Glu 133, Asn 140, Trp 141, Gly 142, Val 145, Phe 195, Leu 198, Tyr 199	Ala 97, Phe 101, Met 112, Val 130, Leu 134, Arg 143, Ala 146, Phe 150	1.20
**Compound**	**Mcl1-1**			
	**ΔG** **(kcal/mol)**	**H-BR**	**NPI**	**RMSD**
Geranylgeraniol	−6.46	Val 253, Thr 266, Leu 267, Ile 268, Gly 271, Ile 294	Ala 227, Phe 228, Met 231, Leu 235, Leu 246, Val 249, Met 250, Phe 270, Val 274, Leu 290	-
Phytol	−6.35	Phe 228, Arg 263, Thr 266, Gly 271, Val 274, Ile 294	Met 231, Leu 235, Leu 246, Val 249, Met 250, Val 253, Phe 254, Leu 267, Phe 270, Leu 290	-
Farnesyl acetate	−6.69	Val 253, Phe 254, Arg 263, Thr 266, Gly 271	Met 231, Leu 235, Leu 246, Val 249, Met 250, Leu 267, Phe 270, Val 274, Leu 290, Ile 294	-
9EA	−10.77	Val 216, Phe 228, Leu 235, Val 249, Phe 254, Gly 262, Arg 263, Val 265, Gly 271, Leu 290, Ile 294, Phe 318, Phe 319	Val 220, His 224, Ala 227, Met 231, Leu 246, Met 250, Val 253, Thr 266, Leu 267, Phe 270, Val 274	1.45
**Compound**	**VEGFR-2**			
	**ΔG** **(kcal/mol)**	**H-BR**	**NPI**	**RMSD**
Geranylgeraniol	−6.3	Glu 885, Phe 918, Cys 919, Lys 920, Gly 922, Ile 1044, Asp 1046	Leu 840, Val 848, Ala 866, Lys 868, Leu 889, Val 898, Val 899, Val 914, Val 916, Leu 1035, Cys 1045, Phe 1047	-
Phytol	−5.98	Lys 868, Val 898, Phe 918, Cys 919, Lys 920, Gly 922, Asn 923, His 1026, Ile 1044, Asp 1046	Leu 840, Val 848, Ala 866, Leu 889, Val 899, Val 914, Val 916, Leu 1035, Cys 1045, Phe 1047	-
Farnesyl acetate	−5.82	Leu 840, Ala 866, Phe 918, Cys 919, Gly 922, Ile 1044, Asp 1046	Val 848, Lys 868, Leu 889, Val 899, Val 916, Leu 1035, Cys 1045, Phe 1047	-
Axitinib (AG-013736)	−8.57	Glu 885, Leu 889, Glu 917, Phe 918, Cys 919, Gly 922, Asn 923, Asp 1046	Leu 840, Val 848, Ala 866, Lys 868, Val 899, Val 914, Val 916, Cys 919, Leu 1035, Cys 1045, Phe 1047	1.8

ΔG: binding energy (kcal/mol); H-BR: H-binding residues; NPI: nonpolar interactions; Ala: alanine; Asp: aspartate; Asn: asparagine; Arg: arginine; Cys: cysteine; Lys: lysine; Thr: threonine; Trp: tryptophan; Leu: leucine; His: histidine; Gly: glycine; Glu: glutamic acid; Ile: isoleucine; Tyr: tyrosine; Phe: phenylalanine. All results were obtained through docking approaches.

## Data Availability

The data presented or additional data in this study are available on request from the corresponding author.

## Supplementary Materials

The following supporting information can be downloaded at: https://www.mdpi.com/article/10.3390/molecules29091956/s1, Figure S1. Molecular Docking of the terpenoids bound to Bcl-2, (**A**) geranylgeraniol, (**B**) phytol, (**C**) farnesyl acetate and (**D**) navitoclax. Figure S2. Molecular Docking of the terpenoids bound to Bcl-2, (**A**) geranylgeraniol, (**B**) phytol, (**C**) farnesyl acetate and (**D**) navitoclax. Figure S3. Molecular Docking of the terpenoids bound to VEGFR-2, (**A**) geranylgeraniol, (**B**) farnesyl acetate, (**C**) phytol and (**D**) axitinib.
