# Supplementary material for: In Vivo, In Vitro and In Silico Anticancer Activity of Ilama Leaves: An Edible and Medicinal Plant in Mexico
Source: Molecules. 2024 Apr 24;29(9):1956. doi: 10.3390/molecules29091956 (PMC11085222; doi:10.3390/molecules29091956)
Supplement: Supplementary file 1 [file molecules-29-01956-s001.zip › molecules-2953899-supplementary.pdf]

In vivo, in vitro and in silico Anticancer Activity of Ilama Leaves. An Edible and Medicinal Plant in Mexico.

Jesica Ramírez-Santos [1,2], Fernando Calzada [2,*], Rosa María Ordoñez-Razo [3], Jessica Elena Mendieta-Wejebe [1], José Antonio Velázquez-Domínguez[4], Raúl Argüello-García[5], Claudia Velázquez[6], and Elizabeth Barbosa[1]

Supplementary material

Figure S1. Molecular Docking of the terpenoids bound to Bcl-2, (**A**) geranylgeraniol, (**B**) phytol, (**C**) farnesyl acetate and (**D**) navitoclax.

Figure S2. Molecular Docking of the terpenoids bound to Mcl-1, (**A**) geranylgeraniol, (**B**) phytol, (**C**) farnesyl acetate and (**D**) 9EA

Figure S3. Molecular Docking of the terpenoids bound to VEGFR-2, (**A**) geranylgeraniol, (**B**) farnesyl acetate, (**C**) phytol and (**D**) axitinib.

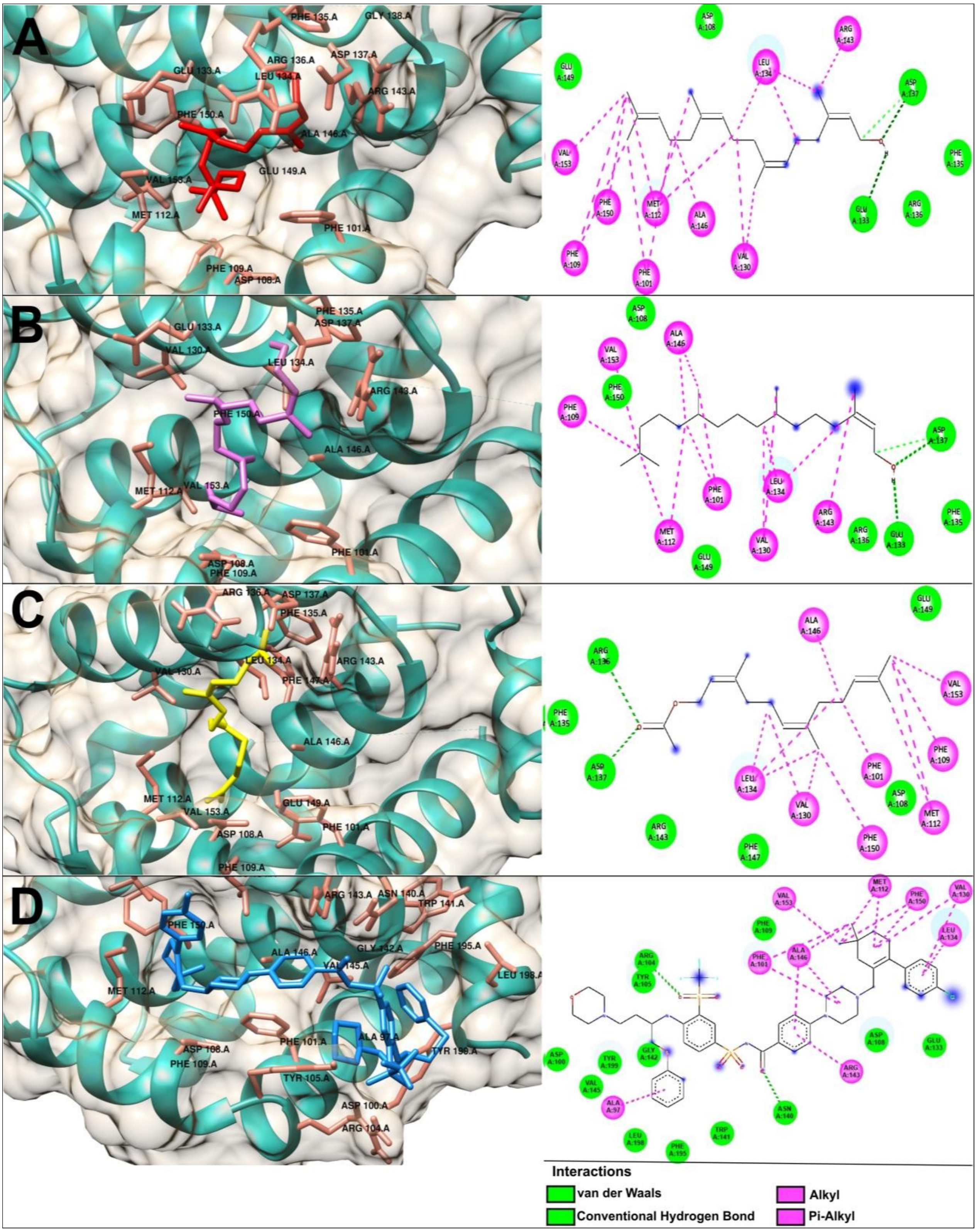

Figure S1. Molecular Docking of the terpenoids bound to Bcl-2, (**A**) geranylgeraniol, (**B**) phytol, (**C**) farnesyl acetate and (**D**) navitoclax

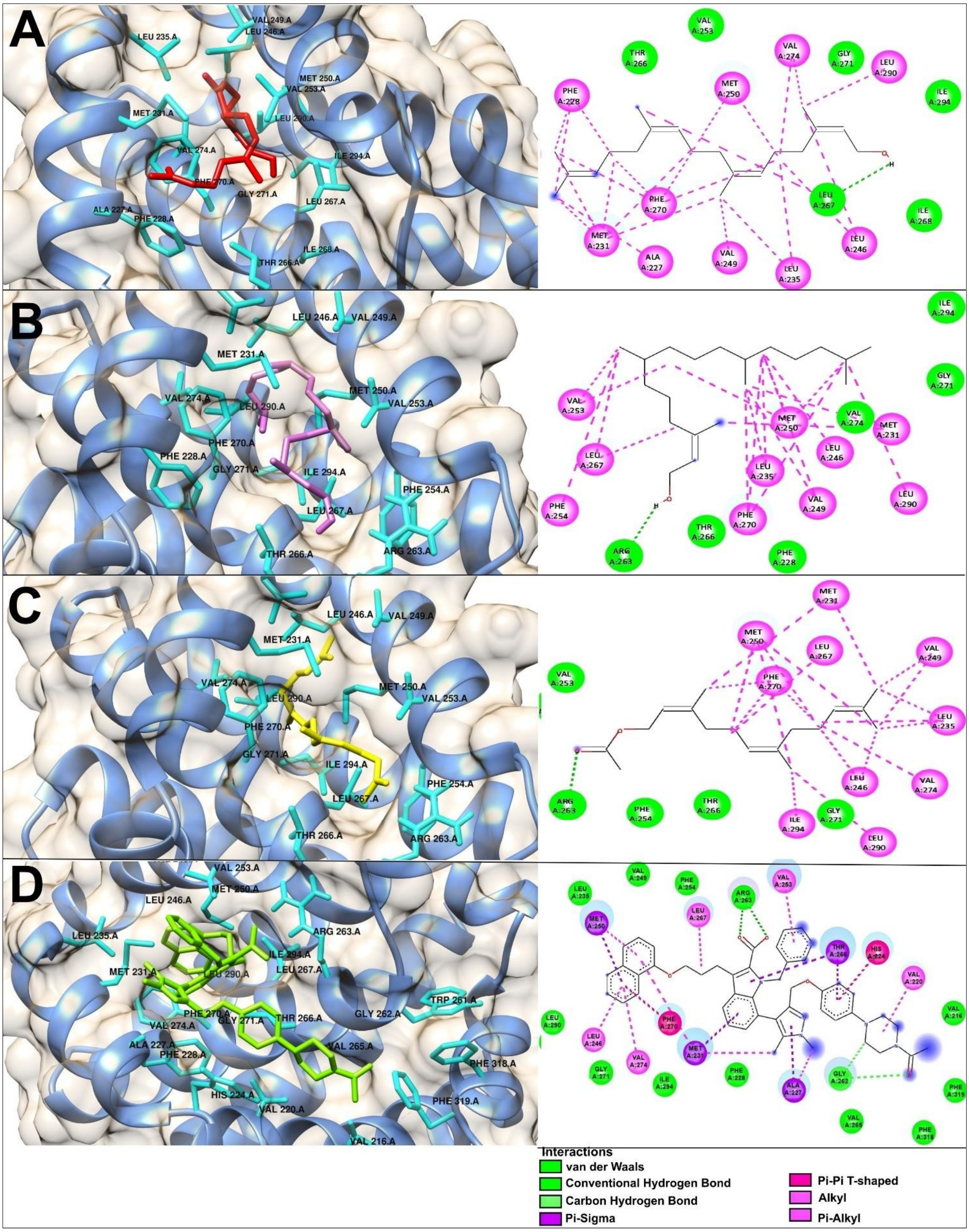

Figure S2. Molecular Docking of the terpenoids bound to Mcl-1, (**A**) geranylgeraniol, (**B**) phytol, (**C**) farnesyl acetate and (**D**) 9EA.

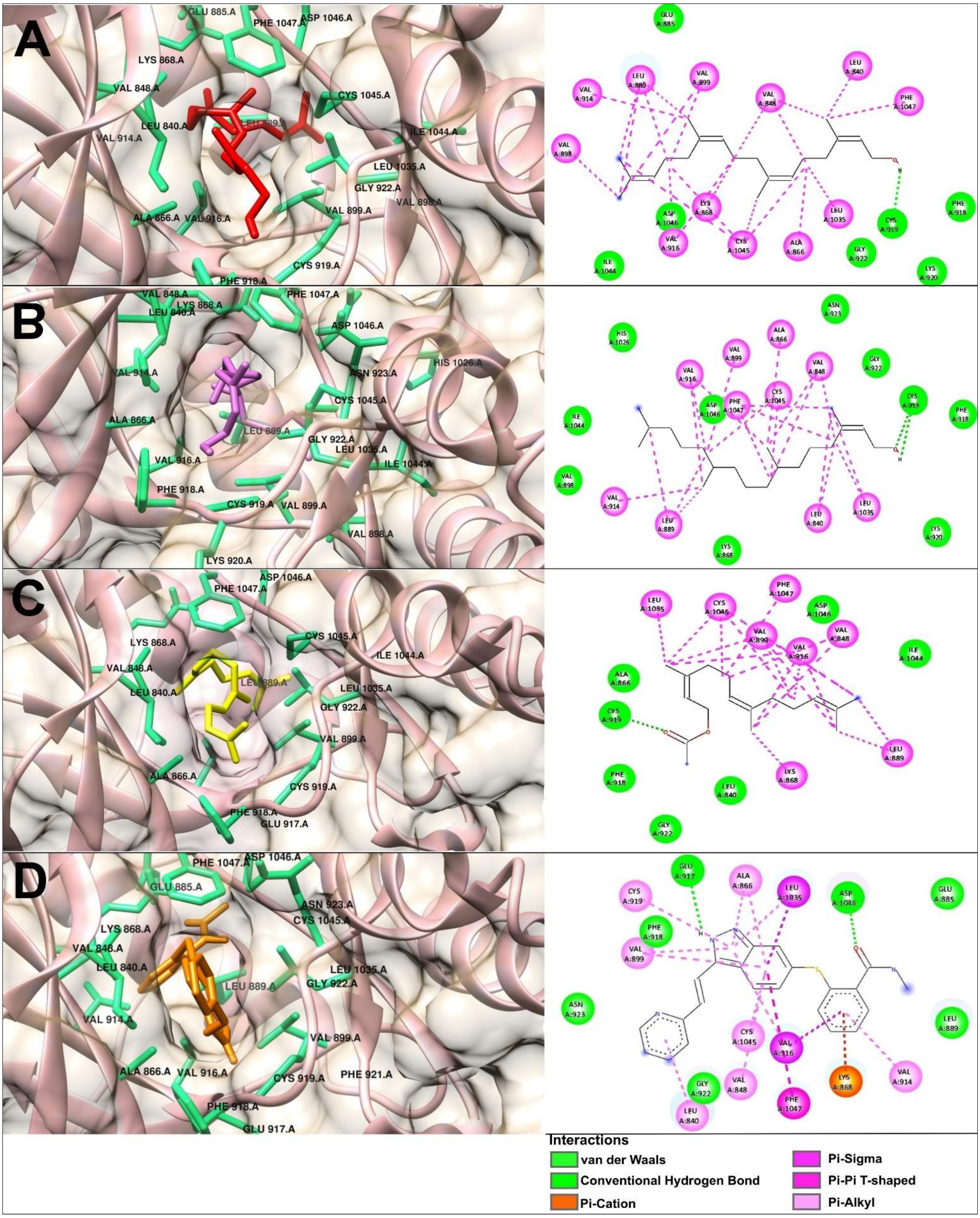

Figure S3. Molecular Docking of the terpenoids bound to VEGFR-2, (**A**) geranylgeraniol, (**B**) farnesyl acetate, (**C**) phytol and (**D**) axitinib.
